# An Australian general practice based strategy to improve chronic disease prevention, and its impact on patient reported outcomes: evaluation of the preventive evidence into practice cluster randomised controlled trial

**DOI:** 10.1186/s12913-017-2586-4

**Published:** 2017-09-08

**Authors:** Mark Fort Harris, Sharon M. Parker, John Litt, Mieke van Driel, Grant Russell, Danielle Mazza, Upali W. Jayasinghe, Jane Smith, Chris Del Mar, Riki Lane, Elizabeth Denney-Wilson, On behalf of the Preventive Evidence into Practice Partnership Group

**Affiliations:** 1 Centre for Primary Health Care and Equity, University of New South Wales, Sydney, 2052 NSW Australia; 2 Discipline of General Practice, Health Sciences Building, Flinders University, Adelaide, 5042 SA Australia; 3 Academic Discipline of General Practice, School of Medicine, University of Queensland, Brisbane, 4072 QLD Australia; 4 Southern Academic Primary Care Research Unit, Monash University, Melbourne, 3800 VIC Australia; 5 Department of General Practice, School of Primary Health Care, Monash University, Melbourne, 3800 VIC Australia; 6 Faculty of Health Sciences and Medicine, Bond University, Gold Coast, 4229 QLD Australia; 7 Health Sciences and Medicine, Bond University, Gold Coast, 4229 QLD Australia; 8 Sydney Nursing School and Sydney Local Health District, The University of Sydney , Sydney, 2006 NSW Australia

**Keywords:** Primary health care, Family practice, Primary prevention, Delivery of health care, Quality improvement; practice guidelines, Evidence based medicine, Cardiovascular disease

## Abstract

**Background:**

Implementing evidence-based chronic disease prevention with a practice-wide population is challenging in primary care.

**Methods:**

PEP Intervention practices received education, clinical audit and feedback and practice facilitation.

Patients (40‑69 years) without chronic disease from trial and control practices were invited to participate in baseline and 12 month follow up questionnaires.

Patient-recalled receipt of GP services and referral, and the proportion of patients at risk were compared over time and between intervention and control groups. Mean difference in BMI, diet and physical activity between baseline and follow up were calculated and compared using a paired t-test. Change in the proportion of patients meeting the definition for physical activity diet and weight risk was calculated using McNemar’s test and multilevel analysis was used to determine the effect of the intervention on follow-up scores.

**Results:**

Five hundred eighty nine patients completed both questionnaires. No significant changes were found in the proportion of patients reporting a BP, cholesterol, glucose or weight check in either group. Less than one in six at-risk patients reported receiving lifestyle advice or referral at baseline with little change at follow up. More intervention patients reported attempts to improve their diet and reduce weight. Mean score improved for diet in the intervention group (*p* = 0.04) but self-reported BMI and PA risk did not significantly change in either group. There was no significant change in the proportion of patients who reported being at-risk for diet, PA or weight, and no changes in PA, diet and BMI in multilevel linear regression adjusted for patient age, sex, practice size and state. There was good fidelity to the intervention but practices varied in their capacity to address changes.

**Conclusions:**

The lack of measurable effect within this trial may be attributable to the complexities around behaviour change and/or system change. This trial highlights some of the challenges in providing suitable chronic disease preventive interventions which are both scalable to whole practice populations and meet the needs of diverse practice structures.

**Trial registration:**

Australian and New Zealand Clinical Trials Registry (ANZCTR): ACTRN12612000578808 (29/5/2012). This trial registration is retrospective as our first patient returned their consent on the 21/5/2012. Patient recruitment was ongoing until 31/10/2012.

## Background

The provision of effective preventive care aims to reduce preventable morbidity and mortality, enhance quality of life and decrease an individual’s need generally for medical services [1]. As people age, having favourable risk factor levels not only decreases the lifetime risk of developing a chronic illness, it also increases survival should a chronic condition occur [2]. Primary care can provide a routine range of preventive activities including opportunistic screening and risk factor identification [3], and general practitioners (GPs) can positively influence their patient’s lifestyle choices, and encourage and equip them to take a greater interest in, and greater responsibility for, their own health [4]. In practice, however, few primary care encounters in Australia involve risk-factor assessment and intervention [5].

Australian health policy encourages general practice to routinely incorporate prevention [6, 7] via a range of initiatives provided through the national health insurance scheme, Medicare. These fund or incentivise primary care-based prevention activities including health checks for specific age groups and at-risk populations, and the utilisation of practice nurses (PNs) to conduct health assessments [8].

Clinical guidelines which provide evidence based recommendations for clinical management and early identification and management of biomedical risk factors such as obesity, high blood pressure, high blood cholesterol and impaired glucose tolerance further support general practice to undertake prevention [9–11]. Although GPs generally accept such guidelines, significant barriers have been identified to the uptake and implementation of these [12–14].

Patients also report some barriers when seeking preventive care such as limited consultation time, a focus by the GP on acute health care issues as opposed to prevention, and receipt of superficial preventive advice without sufficient follow up or exploration of the personal or behavioural mechanisms associated with continued risk behaviour [15].

Strategies that have been found to effectively aid the implementation of guidelines in general practice include small group education for providers, clinician prompts and decision aids, audit and feedback and external facilitation [16]. Practice facilitation is a supportive service utilising a range of organisational development, project management, quality improvement (QI), and practice improvement approaches to build internal capacity, enabling the engagement in activities which provide support over time to achieve incremental and transformative improvement goals [17]. Practice facilitation is a promising method to overcome organisational or system level barriers to evidence translation [18] where trained external facilitators, not involved in direct patient care, are used to support practices to implement changes that will improve the quality of the care they provide [19, 20]. The advantage of practice facilitation is that it allows programs to be tailored to individual practice situations [21] and it supports practice redesign [22], resulting in more sustainable work practices [21] and increased internal capacity [20]. A systematic review by Baskerville, found primary care practices to be more than twice as likely to adopt evidence-based guidelines through practice facilitation [18]. There is also some evidence that practice facilitation leads to improvements in quality of care [22] and levels of preventive care [21].

The Preventive Evidence into Practice (PEP) study was a cluster randomised trial implemented concurrently in four Australian states during 2012/2013. The trial evaluated the effectiveness of a model of education, clinical audit and feedback and practice facilitation that aimed to improve the implementation of vascular disease prevention guidelines in Australian general practice. Previously published data from this trial includes clinical record audit data for 21,848 patients and survey data from general practitioners (GPs) and practice nurses. Outcomes reported include recording rates for smoking, alcohol intake, body mass index (BMI), waist circumference and blood pressure for deidentified patients aged 45–69 years and lipids, fasting blood glucose and cardiovascular risk for those aged 40–69 years; and change in self-reported frequency and confidence of clinicians in conducting assessments for their patients [23].

The aim of this paper is to provide base-line and 12 month questionnaire data for patients who were recruited from general practices participating in the PEP trial.

## Methods

### Design, eligibility and recruitment

The trial design has been previously reported [24]. Sixteen practices were assigned to receive the PEP intervention and 16 provided usual care. A random sample of 160 patients from each practice was identified using PenCat clinical audit software. Eligible patients were 40–69 years without known diabetes, cardiovascular disease or renal impairment, and who had visited a study practice within the last year. Practice staff vetted the patient list to ensure that only those patients with proficient English and cognitive awareness were approached. Additional exclusion criteria included severe mental illness, substance abuse or pregnancy. Eligible patients received a mailed invitation, and those willing to participate, returned their consent to the PEP study centre at the University of New South Wales. Study questionnaires were mailed at baseline and 12 months.

### The PEP practice intervention

The PEP trial was designed to address known evidence-practice gaps in chronic disease prevention in general practice. These include substantial undertreatment and low rates of appropriate treatment in patients at high risk of a cardiovascular event [25, 26]; poor recording of BMI and waist circumference [27] and suboptimal assessment of lifestyle risk factors including smoking status, alcohol intake, diet and weight [28]. Other gaps include appropriate advice and referral to deal with lifestyle risk. Research suggest that less than a third of overweight and obese patients in general practice receive some form of lifestyle assistance (diet or exercise) for weight loss from their GP [29] and referral rates for educational or behavioural interventions are generally low [30, 31].

**Fig. 1:**
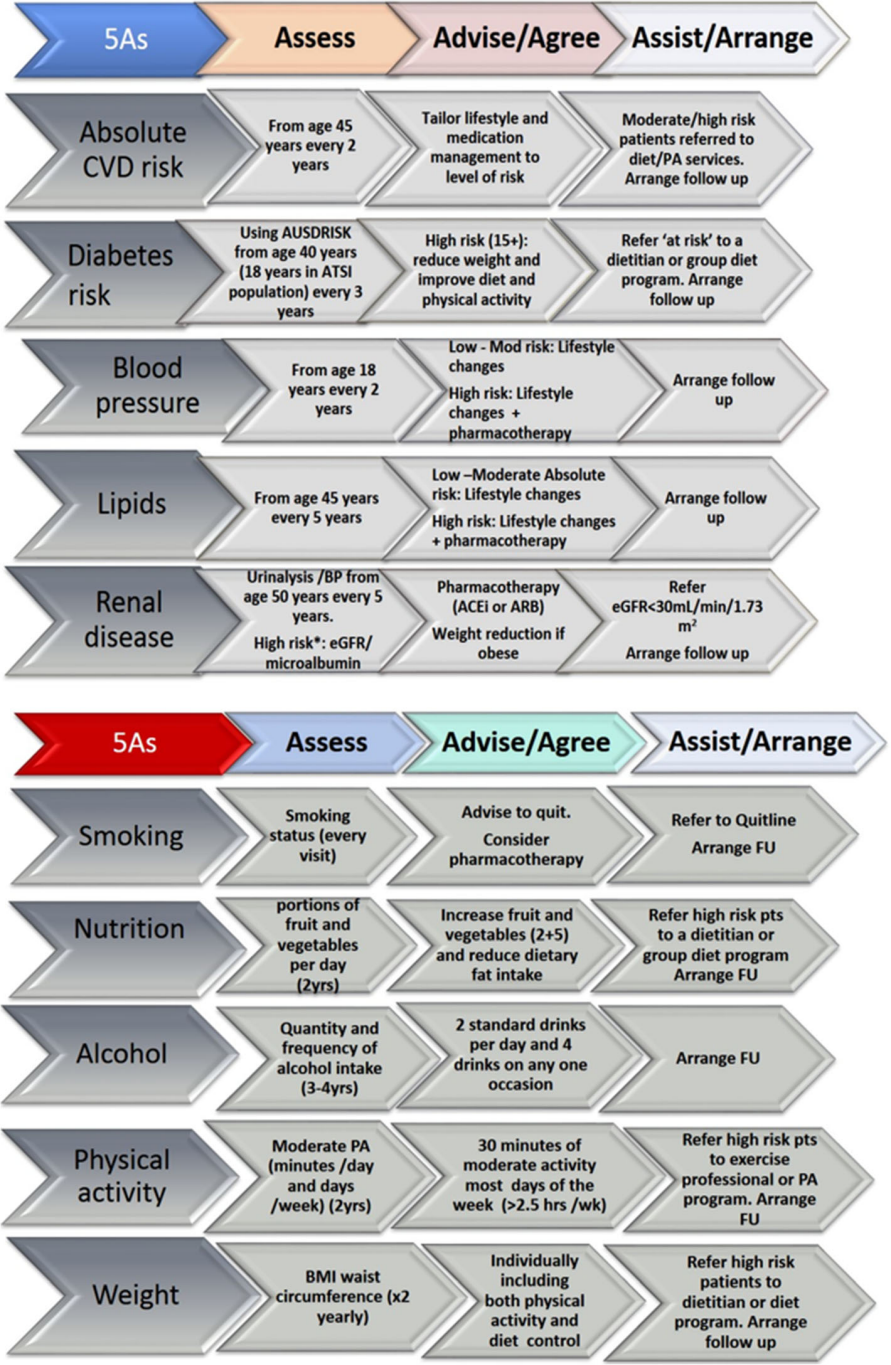
PEP prevention Reference Guide

The practice level intervention targeted the clinical management of patients by both GPs and PNs and aimed to promote evidence based preventive care of patients who smoke, drink excessive alcohol, have a poor diet, are physically inactive, are obese or have a risk of cardiovascular disease or diabetes. A synthesis of major guideline recommendations for the prevention of vascular disease from the National Vascular Disease Prevention Alliance (NVDPA) [11], the Royal Australian College of General Practitioners (RACGP) [9] and the National Health and Medical Research Council (NHMRC) [10] of Australia were synthesise across the 5As framework and provided to each intervention practice as a quick reference guide (Fig. 1).

This framework (Assess, advise/Agree, Assist/Arrange) provides a brief intervention model for the delivery of preventive care in primary health care including the process of behaviour change for patients [24]. This framework has been incorporated into a range of clinical practice guidelines [25], and the guidance for clinical care and organisation of general practice in relation to behavioural risk factors by the Royal Australian College of General Practitioners (RACGP) [9].

The intervention comprised the following:

1. Clinician education – Clinicians received three hours of interactive, small group education. At these sessions, the preventive evidence reference guide was provided with presentations as to the recommendations within the guidelines. The education sessions also explored aspects of readiness to change and sought to equip the clinicians with specific skills in motivational interviewing and brief behaviour change techniques with their patients.
2. Audit and feedback – practices underwent a de-identified clinical audit of their data and received feedback reports which highlighted issues with recording and provided recommendations to improve this.
3. Practice facilitation visits –Intervention Facilitators (IFs) were sourced from local primary care organisations (Medicare Local) where they had roles undertaking QI activities with general practice. Facilitators underwent a 1 day training session with the study investigators and were supervised and supported through the intervention by the study investigators and study coordinator. Facilitators used each practice’s clinical audit report to identify specific areas of need for the practice and to identify organisational, clinical, and business functions of the practice that could be improved to support improved preventive care (i.e. changes/clarification to staff roles and activities). They assisted with the identification of a ‘practice prevention coordinator’ and other changes to practice systems and resources (e.g. use of assessment tools, correct recording and ordering of specific education materials). A locally relevant information directory was also provided to practices comprising associated community services, providers or programs (e.g. phone advice lines, exercise programs). The facilitator reviewed usual practice and advised about Medicare-funded items that support preventive care visits, the identification of high risk patients, and systems to support patient recall. Three practice facilitation visits of 1‑2 h each were provided as well as follow up phone calls between visits to keep practices engaged with the facilitation process.

## Study logic and rationale

**Fig. 2:**
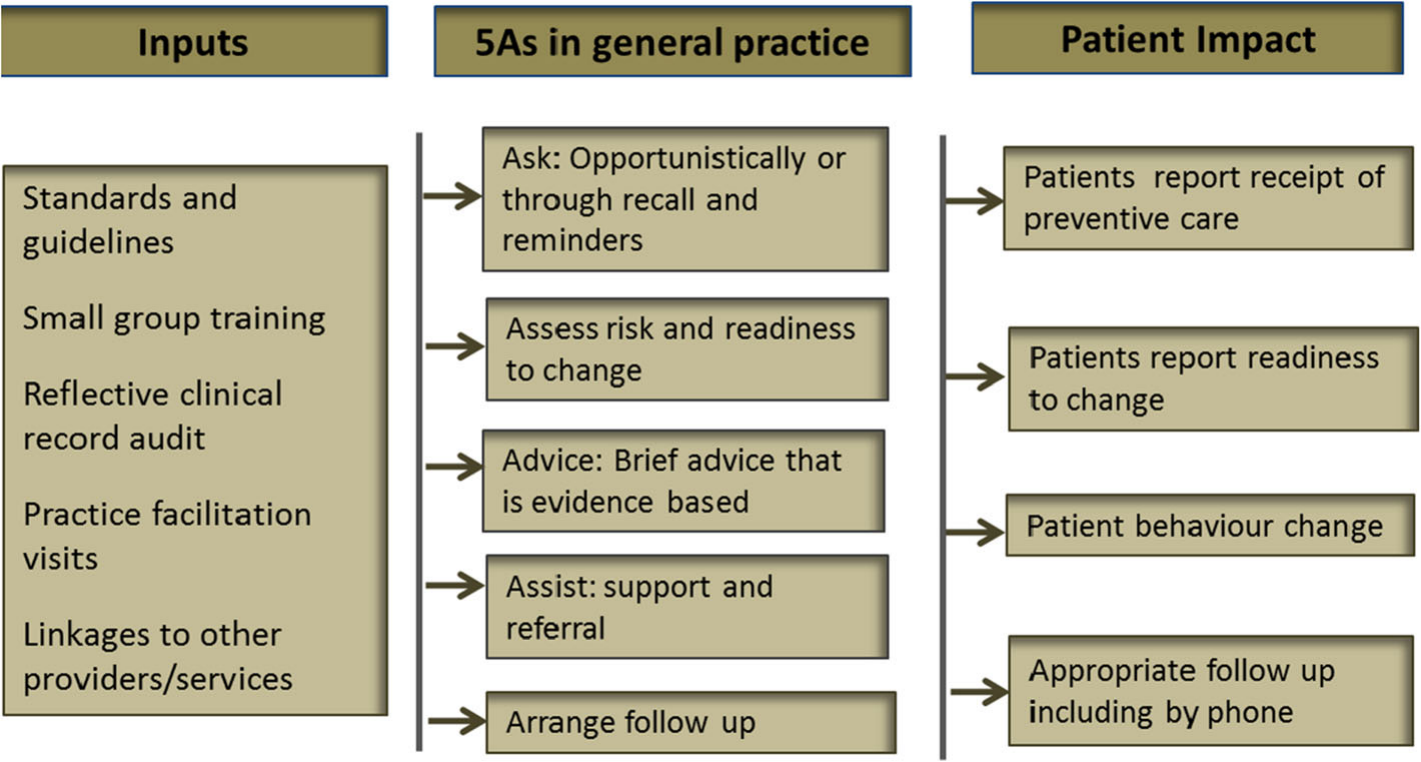
PEP study logic model

The study logic model (Fig. 2) was also developed around the 5As. The main assumption within the model was that increased access to preventive evidence provided via the reference guide and reiterated through the education and facilitation, would encourage clinicians to improve their preventive practices (increased screening and the use of tools to identify at-risk patients; increased recall, referral, recording and better clinical management).

We also hypothesised that this would result in improved patient/provider interaction resulting in a reduction in both risk behaviour and physiological risk over the 12-month period of the trial.

### Study outcomes

Outcomes assessed were all self-reported patient outcomes. These included impacts on lifestyle risk (and stage of readiness to change), impact on preventive care received, use of GP services and level of advice and referral received from their practice.

### Patient data collection instrument and management of data

The PEP patient questionnaire has been used in previous research by the authors [32]. It utilised items from the NSW Adult Heath Survey [33] and the National Health Survey [34]. The content of the NSW Adult Health Survey was initially developed in consultation with NSW Area Health Services (AHS), government departments, and a range of experts. The content was designed to cover the eight health priority areas for NSW (asthma, Diabetes, Cardiovascular health, Mental health, injury prevention, arthritis and musculoskeletal conditions, Dementia) [35] and was translated into a number of languages. The National Health Survey is designed to obtain national benchmark information on a range of health-related issues and to enable the monitoring of trends in health over time [34] and is regularly administered by the Australian Bureau of Statistics (ABS). Other validated instruments were also incorporated within the PEP questionnaire to measure health literacy (Health Literacy Management Scale) [36] and health related quality of life (SF12) [37]. These outcomes have been reported separately [38].

The questionnaire asked patients for personal demographic information and self-reported information about diet, smoking and levels of PA, and attempts to change these within the last 3 months. Respondents were asked to recall whether they had received preventive care in the 3 months prior to the data collection time points. This included the total number of visits to their GP, the number of health checks undertaken and whether blood pressure, blood cholesterol and blood sugar and weight had been assessed during consultation. Patients also reported any advice they recalled being given by their GP concerning alcohol use, smoking cessation, healthy eating, weight control, elevated cholesterol or blood pressure and general prevention of diabetes and heart disease. Information on referral to attend lifestyle related health programs for any of these issues was also collected, as was patient reported attendance due to referral.

We used the Transtheoretical Model of Change [39] to categorise patient ‘readiness to change’. Patients self-reported across six categories of health-related behaviour: eating more fruit and vegetables; eating less dietary fat; doing more physical activity; drinking less alcohol; losing weight and stopping smoking. Their responses were assessed against the stages of ‘pre-contemplation’, ‘contemplation’, ‘action’ and ‘maintenance’ [40], Contemplation was intent to change behaviour within the next 1‑6 month period, action included those who were currently taking measures to change, and maintenance were those who had already changed their behaviour, and had maintained this change for the previous 6 months.

Smoking and alcohol consumption were assessed across two categories (smoker/non-smoker; and 2 or less/more than 2 standard drinks per day). A PA score was calculated based on the weekly frequency of doing vigorous and/or moderate physical activity and a range for individuals was calculated (from 0 to 8) with a score of four or less considered at-risk [41]. Diet score was the number of serves of fruit and vegetables consumed in an average day with six or less serves being considered at-risk [10], and BMI was calculated from patient reported weight and height with at-risk being 25 and higher [11].

Patient questionnaires were checked for consistency and logged at the University of New South Wales (UNSW) study centre before being externally scanned. Data sets underwent cleaning and re-coding and the data belonging to participants who had completed the questionnaire at both timepoints were matched. Analysis was undertaken using SPSS Statistics 22.

## Analysis

### Patient questionnaire

Patient demographic characteristics were assessed for variability at baseline. Further analysis was conducted on 589 patients who completed baseline and follow up questionnaires.

We compared change over time and change between intervention and control groups at baseline and 12 months for patient-recalled receipt of GP services and referral. In assessing ‘readiness to change’, the proportion of patients in the action and maintenance categories with risk factors was compared between intervention and control at baseline and at 12 months. Data was missing for 1–2% of questions (with no differences between intervention and control groups). Patients with missing data were included in the denominator for calculating risk rates.

For BMI, diet and PA scores, mean differences between baseline and follow up were calculated and compared using a paired t-test. Change in the proportion of patients meeting the definition for PA, diet and weight risk was calculated using McNemar’s test and compared between intervention and control groups at both time points. Multilevel analysis was performed using MLwiN version 2.25 [42] to determine the effect of the intervention on follow up scores after adjustment for baseline scores and other covariates. Two-sided *p*-values of less than 0.05 were considered statistically significant.

### Assessing fidelity of the PEP intervention

The PEP intervention was a multi-component intervention delivered at the practice level. When conducting research in general practice there are several ‘real world’ contextual issues that make the delivery of a controlled intervention challenging. Practices vary in their structure and processes, and the available time to undertake research. Each will have different levels of motivation to participate in research and each clinician will have a different level of interest and skill in delivering research based activities. Within this study, we assessed if the components of the PEP intervention were administered to practices (i.e. as per study protocol). This included the number of practices where enrolled clinicians attended the education sessions and the number of practices that participated in facilitation visits and follow up phone calls. This was tabulated and we calculated the mean length (in weeks) that it took to implement the facilitation component of the intervention to practices in each state and compared these across the 4 states.

Qualitative interviews with facilitators were used to assess if there were variations within the facilitation processes, to gauge their experiences working with practices, and any factors that they perceived to have impacted on the practices ability to implement the PEP intervention. Each facilitator kept a record (diary) of each practice visit and phone call. These reports documented short and long-term practice goals and relevant activities undertaken at the practice via a template structured around the chronic care model. This also provided opportunities to document perceived challenges and barriers to change within each practice.

Qualitative interviews conducted with GPs and PNs about the facilitation process were used to enhance our understanding around the patient responses particularly in relation to the preventive care received from their GP. This interview data was collected in NVivo and common factors and themes were drawn out and summarised. Feedback from GPs from the process used to allocate professional development points for the activity was also reviewed to ascertain the perceived quality of care that GPs felt they were providing, including their specific learnings from the project.

## Results

### Fidelity of the intervention

There was reasonable fidelity to the PEP intervention determined by practice participation in education and facilitation sessions (Table 1). All intervention practices apart from the one excluded practice (Victoria), participated in the clinical education sessions. Training was not always achieved through the state-based project workshops. In some instances, supplementary education sessions were provided as group sessions at individual practices, or on a one-to one basis.

**Table 1:** Participation in the PEP intervention by intervention practices

	NSW	Victoria	South Australia	Queensland
Education sessions for GPs and PNs	5/5 practices	4/5 practices^a^	3/3 practices	3/3 practices
% of practices receiving all 3 facilitator visits	100%	80%^a^	100%	100%
% of practices receiving all facilitator phone calls	100%	80%^a^	100%	67%
Mean length (range) of the practice facilitation (in weeks)	9.6 (8–12 weeks)	11.7 (11–13 weeks)	17 (13–19 weeks)	17.5 (17–18 weeks)

^a^1 practice excluded due to *IT* incompatibility issue

The full complement of facilitator visits (*n* = 3) and follow up phone calls were delivered across all practices except in one state (Queensland) where facilitators were not able to contact any of the three intervention practices for the first phone call. We planned to provide the intervention over a 12-week period, but in fact the length of the facilitation ranged from eight to nineteen weeks. In those practices where the facilitation took longer, this was due to several factors ranging from non-availability of clinical staff, unexpected staff turnover and practice closures over the Christmas holiday period. Data from 46 of the 70 participating GPs (66%) indicated that the learning component of the study was generally well received by GPs with more GPs (70%) than not (30%) reporting that their learning objectives and learning needs had been met by the study.

### Impact on practice organisation

The objectives identified during facilitation were very similar with no overt variance noted by state or facilitator. Due to sub-optimal levels of recording identified from many of the practice audits, a major goal identified by many practices was the improved assessment and recording of height, weight, BMI, waist circumference, alcohol use, smoking status and diabetes risk through administration of the AUSDRISK (Australian Type 2 Diabetes risk assessment tool). Some practices also aimed to increase the use of the absolute cardiovascular risk tool, and one practice aimed for a monthly focus on lifestyle change and the establishment of a walking group.

GPs reported that the audit had heightened their awareness of the need to screen their patients between 40‑69 years more thoroughly, and to improve their systems to correctly identify those patients requiring preventive care. Many had noticed shortfalls in their recording particularly around alcohol intake and smoking status, and had recognised a need to ask patients about their substance use, and provide opportunistic intervention where they could. Many practices recognised that they were doing preventive care in an unsystematic or haphazard way and not using their systems to their full potential. Many GPs said they had been prompted by the study to make better use of recall systems, appointment systems, reminders and prompts, and to think of other ways to incorporate preventive evidence into routine practice. Some practices could make simple changes that quickly rectified recording problems. Other practices had begun to make much larger changes including clinical screening by practice nurses, 40‑49 yr. health checks, and commencement of clinics catering for 40‑49 yr. old patients. System changes such as combining their patient data base with their appointment systems and increased referral to allied health professionals were also common. Tools such as the AUSDRISK and cardiovascular risk calculators were being used more routinely because of the study.

### Baseline patient characteristics

Two practices were lost prior to baseline assessment: one intervention practice was excluded because of incompatibility between the electronic medical record and the data extraction program; and one control practice withdrew citing lack of capacity to continue participating in the study.

**Fig. 3:**
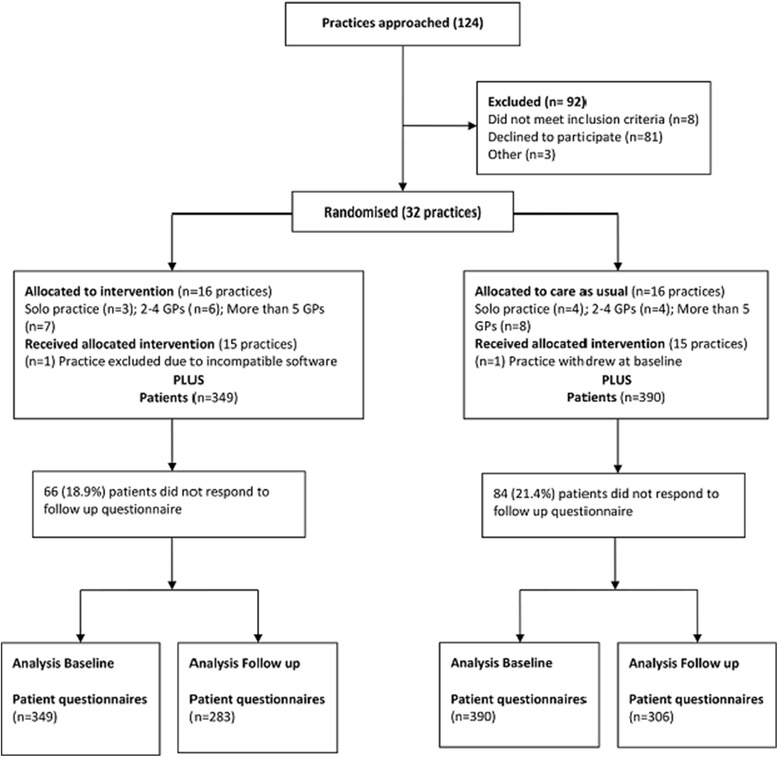
PEP CONSORT flow diagram

The patient sample was recruited from 30 practices (15 intervention; 15 control). Fifteen percent (15%; 349 intervention; 390 control) of all patients invited to participate consented at baseline. Of these, 589 (80%) also completed the 12-month questionnaire (Fig. 3). We found no significant differences between the respondents in the intervention and control practices at baseline in relation to gender, age and other demographic variables (Table 2).

**Table 2:** Patient self-reported baseline characteristics

Baseline patient characteristics	Intervention N (%)	Control N (%)	Significance X^2^
No of patients	349 (47)	390 (53)	
Male	114 (33)	111 (28)	NS
Female	235 (67)	279 (71)	
39yrs^a^	1	0	NS
40-49 yrs	93 (27)	101 (26)	
50-59 yrs	118 (34	150 (38)	
60-69 yrs	133 (38)	135 (35)	
70yrs^a^	1	2	
Missing	3	2	
Mean age	55.6 yrs	55.5 yrs	
English spoken at home	330 (95)	375 (96)	NS
Born in Australia	263 (75)	289 (74)	NS
University degree or higher	119 (34)	141 (36)	NS
Technical/Professional qualification	96 (28)	99 (25)	
School leaving	123 (35)	140 (36)	
Other/not specified	9 (2)	10 (3)	
Missing	2	0	
Employed	232 (66)	249 (64)	NS
Retired	69 (20)	72 (18)	
Unable to work due to illness/disability	11 (3)	18 (5)	
Looking after home/family	21 (6)	32 (8)	
Unemployed	4 (1)	9 (2)	
Full time education	0	3	
Other	6	1	
Missing	6	6	

^a^Three patients were outside the specified age range possibly due to an error in recording at the practice

Respondents were more likely to be female (514 women versus 225 men). Most respondents were born in Australia (75%) and spoke English at home (95%), and were generally skilled, with 62% having a professional/technical or higher university qualification, and 65% being in some form of employment.

### Patient outcomes

1. **Patient self-reported impact on behaviour change**
  Most of those at-risk indicated they were ready to change their behaviours. There was an increase in the proportion of intervention patients in the ‘action’ or ‘maintenance’ stage of readiness to change for eating more fruits and vegetables (from 76.7 to 82.8%), eating less fat (from 71.3 to 80.4%), and losing weight (from 69.7 to 79.5%) (Table 3). There was no significant change for patients in control practices. The proportion of patients reporting readiness to change in relation to risky drinking increased by approximately 40% in both groups but this was based on low baseline numbers.
  When we assessed changes in mean score for PA, diet and BMI risk, only the diet score improved in the intervention group (*p* = 0.04). Self-reported BMI and PA risk however did not significantly change between baseline and follow up in either group (Table 4). There was also no significant change in the proportion of patients who reported being at-risk for diet, PA or weight (Table 5) and no changes in patient reported PA, diet and BMI in multilevel linear regression adjusted for patient age, sex, practice size and state. Data was missing for 1–2% of these questions (no differences between intervention and control groups).
2. **Patient self-reported impact on preventative care received**
  Patient reported preventive care was assessed for the 3 months prior to both baseline and follow up. The follow up assessment took place between 3 and 6 months after the completion of the intervention.
  There were no significant changes between baseline and follow-up in either group for the number of patients who reported either attending a GP consultation, or receiving a preventive health check from their GP in the previous 3 months (Table 6). For patients who reported attending their GP in the previous 3 months, there were no significant changes in the proportion reporting a blood pressure, cholesterol, glucose or weight check in either group (Table 7).
  Although most patients at baseline were at increased risk in respect to diet, physical inactivity or weight (78.8, 44.5 and 57.7% respectively), less than one in 6 of these patients reported that they received advice or referral at baseline Tables 8 and 9). This did not significantly change at follow up apart from a decline in healthy eating advice in the control group (Table 8). A smaller proportion of patients at baseline smoked (8.5%) or drank alcohol at risky levels (more than 2 standard drinks in a typical day) (26.2%), and the frequency of advice and referral also did not change significantly for these factors.

**Table 3:** Proportion of patients with risk factors in action or maintenance stage of change^a^

	Intervention N (%; 95% CI)		Control N (%; 95% CI)	
	Baseline	Follow up	Baseline	Follow up
Eat more fruits or vegetables	243 (76.7%; 72.0–81.3)	207 (82.8%; 78.1–87.5)	297 (83.0%; 79.1–86.9)	226 (82.5%; 78.0–87.0)
Eat less dietary fat	226 (71.3%; 66.3–76.3)	201 (80.4%; 75.5–85.3)	279 (77.9%; 73.6–82.2)	207 (75.5%; 70.5–80.6)
Do more physical activity	194 (69.8%; 64.4–75.2)	154 (75.5%; 69.6 81.4)	230 (69.9%; 65.0–74.9)	170 (74.2%; 68.6–79.9)
Lose weight	138 (69.7%; 63.3–76.1)	124 (79.5%; 73.2–85.8)	165 (74.3%; 68.6–80.1)	125 (74.4%; 67.8–81.0)
Less alcohol	9 (9.6%; 3.6–15.5)	36 (52.9%; 41.1–64.8)	9 (9.4%; 3.5–15.2)	34 (50.7%; 38.8–62.7)
Stop smoking	7 (21.9%; 7.6–36.2)	7 (35.0%; 14.1–55.9)	10 (32.3%; 15.8–48.7)	6 (31.6%; 10.7–52.5)

^a^Patients in action stage (taking steps to change behaviour) or maintenance stage (have overcome some barriers to sustained change)

**Table 4:** Changes in mean score for PA, diet and BMI

	Intervention			Control		
Risk factor	Baseline (349)	Follow up (283)		Baseline (390)	Follow up (306)	
	Mean Score (95% CI)	Mean Score (95% CI)	Mean diff	Mean Score (95% CI)	Mean Score (95% CI)	Mean diff
Physical activity	3.67 (3.40–3.98)	3.85(3.56–4.15)	0.18, *t* = 1.4, NS	3.37 (3.13–3.63)	3.47 (3.21–3.73)	0.09, *t* = 08, NS
Diet	4.51 (4.10–4.94)	5.00 (4.76–5.25)	0.49, *t* = 2.5, p = 0.04)	4.87 (4.60–5.14)	5.05 (4.82–5.28)	0.17, *t* = 1.6, NS
BMI	26.40 (25.72-27.10)	26.08 (25.30-26.82)	0.32, *t* = 1.2, NS	26.08 (25.40-26.73)	26.34 (25.67-27.05)	0.26, *t* = 0.8, NS

**Table 5:** Proportion of patients reporting PA, diet and weight risk

Proportion at-risk	Baseline 349 N (%; 95% CI)	Follow up 283 N (%; 95% CI)	Baseline 390 N (%; 95% CI)	Follow up 306 N (%; 95% CI)
Physical Activity risk (<=4)	165 (47.3%; 42.0–52.5)	151 (53.4; 47.5–59.2)	164 (42.1%; 37.2–47.0)	134 (43.8%; 38.2–49.3)
Diet risk (<=6)	270 (77.4%; 73.0–81.8)	220 (77.7%; 72.9–82.6)	313 (80.0%; 76.0–84.0)	231 (75.5%; 70.7–80.3)
Weight risk (BMI > 25)	202 (57.9%; 52.7–63.1)	156 (55.1%; 49.3–60.9)	225 (57.7%; 52.8–62.6)	168 (54.9%; 49.3–60.5)

**Table 6:** Patient self-reported use of general practice services (All patients previous 3 months)

	Intervention N (%; 95% CI)		Control N (%; 95% CI)	
Health service	Baseline (349)	Follow up (283)	Baseline (390)	Follow up (306)
Attended GP visit(s)	255 (73.1%; 68.4–77.7)	212 (74.9%;69.9–80.0)	292 (74.9%; 70.6–79.2)	231 (75.5%; 70.7–80.3)
0 GP visits	97 (27.8%; 23.1–32.5)	71 (25.1%; 20.0–30.1)	103 (26.4%; 22.0–30.8)	75 (24.5%; 19.7–29.3)
1–2 GP visits	184 (52.7%; 47.5–58.0)	162 (57.2%; 51.5–63.0)	227 (58.2%; 53.3–63.1)	177 (57.8%; 52.3–63.4)
3–5 GP visits	56 (16.0%; 12.2–19.9)	40 (14.1%; 10.1–18.2)	51 (13.1%; 9.7–16.4)	45 (14.7%; 10.7–18.7)
6+ GP visits	12 (3.4%; 1.5–5.4)	10 (3.5%; 1.4–5.7)	9 (2.3%; 0.8–3.8)	9 (2.9%; 1.0–4.8)

**Table 7:** Self-reported receipt of health services (previous 3 months) for patients reporting GP visits

	Intervention N (%; 95% CI)		Control N (%; 95% CI)	
Patients reporting GP visits^a^	Baseline (255)	Follow up (212)	Baseline (292)	Follow up (231)
Preventive health check by GP	164 (64.4%; 58.4–70.2)	126 (59.4%; 52.8–66.0)	185 (63.4%; 57.8–68.9)	142 (61.5%; 55.2–67.7)
Any check by GP (BP/cholesterol/sugar/weight)	205 (80.4%; 75.5–85.3)	173 (81.6%; 76.4–86.8)	252 (86.3%; 82.4–90.2)	197 (85.3%; 80.7–89.9)
Blood pressure check by GP	255 (74.2%; 68.7–79.5)	156 (73.6%; 67.7–79.5)	237 (81.2%; 76.7–85.6)	185 (80.1%; 74.9–85.2)
Blood cholesterol check by GP	112 (43.9%; 37.8–50.0)	99 (46.7%; 40.0–53.4)	127 (43.5%; 37.8–49.2)	115 (49.8%; 43.3–56.2)
Blood sugar check by GP	52 (20.4%; 15.4–25.3)	81 (38.2%; 31.7–44.7)	79 (27.1%; 22.0–32.2)	85 (36.8%; 30.6–43.0)
Weight check by GP	52 (20.4%; 15.4–25.3)	55 (25.9%; 22.0–31.8)	79 (27.1%; 22.0–32.2)	58 (25.2%; 19.5–30.7)

^a^Includes only those patients who reported seeing their GP in Table 6

**Table 8:** Patient self-reported frequency of advice received from their GP

	Intervention N (%) (95% CI)		Control N (%, 95% CI)	
Type of advice/referral	Baseline	Follow up	Baseline	Follow up
Healthy eating advice^a^	26 (11.0%; 7.0–15.0)	30 (15.8%; 10.6–21.0)	35 (13.1%; 9.1–17.2)	12 (5.8%; 2.6–9.0)
Physical activity advice	35 (14.4%; 10.0–18.8)	32 (20.5%; 14.2–26.8)	38 (13.5%; 9.5–17.5)	21 (12.0; 7.2–16.8)
Weight advice	25 (16.0%; 10.3–21.8)	16 (12.5%; 6.8–18.2)	34 (19.1%; 13.3–24.9)	16 (12.0%; 6.5–17.6)
Smoking advice	10 (43.5%; 23.2–63.7)	7 (58.3%; 30.4–86.2)	14 (58.3%; 38.6–78.1)	3 (23.1%; 0.2–46.0)
Alcohol advice	8 (50%; 25.5–74.5)	9 (16.4%; 6.6–26.1)	14 (100%)	7 (15.9%; 5.1–26.7)

^a^Comparison between intervention and control: Baseline *X* ^2^ = 0.4, *p* = 0.6; Follow up *X* ^2^ = 9.3, *p* = 0.002

**Table 9:** Patient self-reported frequency of referral received from their GP

	Intervention N (%) (95% CI)		Control N (%, 95% CI)	
Type of advice/referral	Baseline	Follow up	Baseline	Follow up
Healthy eating referral	9 (3.8%; 1.4–6.3)	5 (2.6%; 0.4–4.9)	12 (4.5%; 2.0–7.0)	10 (4.8%; 1.9–7.8)
Physical activity referral	8 (3.3%; 1.0–5.5)	8 (5.1%; 1.7–8.6)	13 (4.6%; 2.2–7.1)	8 (4.6%; 1.5–7.7)
Weight referral	8 (5.1%; 1.7–8.6)	7 (5.5%; 1.5–9.4)	12 (6.7%; 3.1–10.4)	9 (6.8%; 2.5–11.0)
Smoking referral	0	0	2 (8.3%; 0–19.4)	0
Alcohol referral	1 (6.3%; 0–18.1)	1 (1.8%; 0–5.3)	4 (28.65; 4.9–52.2)	1 (2.3%; 0–6.7)

For Tables 8 and 9 patients were those presenting to the GP in the previous 3 months with risk factor present

## Discussion

Many of the practices in this study recognised that they were doing preventive care in an unsystematic or haphazard way. Facilitators reported a wide variation in the organisational ability of practices, and their capacity to address change. During the intervention, most practices had concentrated on improving the assessment and recording of risk factors. Few chose to improve mechanisms to promote patient education or systems to improve referral to lifestyle-related support services. This was duly reflected in the lack of change in patient reported assessment, advice and referral. This may reflect a natural link between recording and assessment and the baseline clinical audit, unduly focusing practices on the deficiencies of their clinical data recording, or it simply may have required less organisational change and was easier to implement than increasing the frequency of advice or referral.

Although evidence supports practice facilitation as an aid to guideline implementation in the primary care setting, the lack of measurable impact found on patient outcomes in this study is not dissimilar to that reported from other studies evaluating practice facilitation [18, 43, 44]. We found no significant difference between the patient groups based on self-reported attendance at general practice, or the recall of preventive care received (preventive health check or measurement of blood pressure, cholesterol, glucose or weight). Despite a focus within the practitioner education on delivering brief behavioural interventions based on preventive guidelines and delivered via the 5As to patients, there was also no difference in the frequency of patient-recalled preventive advice or referral received for lifestyle risk factors in the 3 months prior to baseline or follow up. The impact on the reported clinical management by the GP (assessments, advice and referral) may have been reduced because patients completed the follow up survey at 12 months although the intervention with practice was at 6 months. The frequency of reported rates of advice was however consistent with those reported in other larger population-based Australian studies [29, 45].

This lack of improvement in patient behaviours and outcomes is potentially related to several factors. It was only possible to measure fidelity based on whether practices participated in the education and facilitation sessions as intended. In this respect, there were good levels of participation. What we were unable to measure was the intervention enactment, or the extent to which the clinicians applied what they had learnt through these processes. Although the PEP intervention gave clinicians the resources to conduct brief preventive interventions, we do not know how frequently or effectively these were employed with their patients. We also do not know how well tailored their approach was to patients’ readiness to changed or whether it was sustained over time.

We have confidence in the rationale of the intervention provided to practices, however, the intervention itself was generally of low intensity and delivered over a relatively short time frame (4–6 months). While practices were supportive of the non-prescriptive nature of the intervention which allowed them to choose their own priorities, this did not translate to measurable patient change. This non-prescriptive approach to the clinician component of the intervention likely influenced fidelity and introduced variations in delivery which could not be measured, and may have biased treatment and impacted our interpretation of the results. When introducing behavioural type interventions there are a number of complex factors which are difficult to control [46]. For example, the nature of the patient presentation may have limited the opportunity to employ the 5As or address issues of prevention, time pressures may have cut the sessions short, the willingness of patients and/or clinicians to discuss prevention may have varied from day to day and the competence level of clinicians within the trial may have varied.

Based on 2011 Australian Census data, the patient sample in the PEP trial had both a higher proportion of people with a tertiary education (35% versus 25%) and speaking English at home (95% versus 76.8%) than the general population. This may have positively influenced the respondents’ level of engagement with, and motivation for preventive behaviours [47]. A larger proportion of those ‘at-risk’ at both time points reported being either in the ‘action or maintenance stages’ in relation to diet, PA or weight. Although this provides a useful assessment in terms of readiness to change, it provides limited understanding of the mechanisms that may have been affecting behaviour change in individuals. We found no significant change, for these variables over 12 months in our sample, except for a small increase in the proportion of those in the intervention group who were actively trying to lose weight or eat a better diet. While individual behaviour change is a key vehicle for improving health [48], we know that it does occur gradually and with fluctuations of willingness and motivation [49]. Readiness to change is influenced by an individual’s level of self-efficacy (belief in their ability to change), the specific context, and the patient–practitioner interaction and level of communication [50].

A major strength of this trial is the rigour of the randomised design. Although we recruited patients across all participating practices, the overall response rate to the survey was lower than expected, leaving the trial under-powered to demonstrate small changes in lifestyle risk behaviour. As patient invitation letters from the practices were used to recruit patients we were also unable to compare the characteristics of our sample with the characteristics of those patients who did not respond as we have identifying and clinical information only from consenting patients. It is possible that the patients who chose to respond were more motivated, or more educated than those who did not respond and hence this may have introduced response bias.

Analysis of the baseline demographic data suggests that, compared to other data routinely collected in Australian general practice, people from non-English speaking backgrounds were under-represented within our sample [51] and the level of health literacy among the sample was relatively high [52]. We therefore cannot make accurate judgements as to the representativeness of our sample. It is possible that the level of health literacy among this sample may explain the relatively high numbers reporting that they had initiated change at baseline, and this may have impacted the margin for improvement in behaviour that could reasonably be achieved.

The patient questionnaire relied on self-reported patient response which raises some limitations. Firstly, patients may knowingly under-report behaviours they feel are undesirable such as weight and other risk behaviours [53, 54], or misinterpret what they need to report. Research has shown that self-report of weight is commonly underestimated and height overestimated, hence calculations of BMI based on self-report may be underestimated [55]. Test/retest analysis of health risk factors and self-reported chronic conditions among a sample of people aged 16 years and over from South Australia [56] has also found some variation with self-report of fruit and vegetable intake. This same study states it is reasonable to expect some chance variation for some behavioural related variables such as physical activity, smoking and alcohol consumption. To aid recall accuracy both baseline and follow questionnaires asked for patients to report the preventive care received in the previous 3 months. For follow up this was effectively the care received between months nine and twelve. It is likely that the biggest impact on preventive care would have occurred during and immediately following the intervention and it is therefore possible that the intervention effect could have dissipated by this point. The timing of these types of interventions therefore comparative to their evaluation is a major consideration for the design of interventions which aim to maximise sustainability.

The intervention was also insufficient to change patient health risk behaviours. Patients who are overweight, smoke, drink hazardously and have poor diet or inadequate physical activity generally know that these factors increase their risk of chronic disease, and they may try to change their behaviour a number of times before they are successful [57]. It is recognised that successful behaviour change may require tailored support for people in different social circumstances, at different stages of disease and with different health literacy levels [58]. Changing patient risk behaviours therefore requires a sustained management strategy incorporating individual assessment, goal setting, referral or enrolment in patient education or coaching and ongoing follow up. The lack of these components may therefore contribute to a lack of change in outcomes such as physical activity. Some additional self-management tools and programs may also be beneficial. Within general practice, strategies need to be incorporated into everyday practice routines. Evaluating the interactions between clinicians and patients would be valuable so we can understand why health behaviour interventions work or don’t work and therefore how to design them to be optimally effective and efficient [58].

## Conclusions

We found little impact on patient reported preventive care and risk behaviours from this complex practice level intervention aimed at the adoption of evidence based prevention approaches. Achieving changes in patient behaviour and patient outcomes especially from a practice level intervention is inherently easier for activities such as assessment but much more difficult for advice, goal setting and referral. Practice level interventions are mediated by uncontrollable factors and it is important to carefully monitor changes at the level of the patient-provider encounter. This has implications for quality improvement programs. Our study demonstrates some of the challenges inherent in providing suitable chronic disease preventive interventions in general practice which are both scalable to whole practice populations and meet the needs of diverse practice structures.

## Abbreviations

ABS: Australian Bureau of Statistics
AHS: NSW Area Health Service
BMI: Body Mass Index
GP: General practitioners
IF: Intervention facilitator
PA: Physical activity
PEP: Preventive Evidence into Practice
PN: Practice nurses
QI: Quality improvement
RACGP: The Royal Australian College of General Practitioners
UNSW: University of New South Wales
